# Consumer Online Knowledge-Sharing: Motivations and Outcome

**DOI:** 10.3389/fpsyg.2022.871518

**Published:** 2022-06-03

**Authors:** Yanhe Li, Yanchen Li, Kunshu Ma, Xiu Zhou

**Affiliations:** ^1^School of Business, Macau University of Science and Technology, Macao, Macao SAR, China; ^2^School of Management, Southwest Minzu University, Chengdu, China; ^3^School of Accounting and Finance, Beijing Institute of Technology, Zhuhai, China

**Keywords:** online Q&A reviews, consumer knowledge-sharing, motivations, loyalty, feedback, e-commerce

## Abstract

As a new form of online reviews, Q&A reviews have been recently used by many e-commerce platforms to compensate for the weaknesses and problems related to trust and helpfulness found in traditional online reviews. This research documents what motivates people to share products or purchasing knowledge with others through Q&A reviews and why e-commerce platforms should place an emphasis on Q&A reviews. Importantly, our results provide evidence that, when receiving feedback (i.e., comments and likes), people are more likely willing to share knowledge with others and will have a higher level of loyalty. We believe that this study contributes to knowledge sharing and the e-commerce literature, and also has practical implications.

## Introduction

To ensure the quality of online shopping, consumers often make judgments and decisions through online reviews on e-commerce platforms (Reimer and Benkenstein, 2016). As the main form of electronic word of mouth (eWOM), online reviews can provide consumers with information about products or brands, affect consumers’ frequency of use of e-commerce platforms (Wang et al., 2016) and influence consumers’ attitudes and purchasing behaviors (Kim and Hanssens, 2017).

Online reviews have many advantages; however, they also have some disadvantages. First, online reviews are anonymous and thus less trustworthy. The readers do not know the specific source of reviews and cannot use past experience to judge their quality (King et al., 2014). Moreover, those less reliable review may induce the boomerang effect, which make negative reviews increase consumers’ purchase intentions, but positive reviews reduce these intentions (Reimer and Benkenstein, 2016). Secondly, a review which is useful to one consumer may be useless to others (Genc-Nayebi and Abran, 2017). Online reviews cannot realize the direct interaction between consumers and in turn may not satisfy every individuals’ needs. On the other hand, when there are a large number of product reviews, consumers may be exposed to the problem of overloading and unable to access all the relevant information (Krishnamoorthy, 2015).

To compensate the shortcomings of traditional online reviews, large-scale shopping websites (such as Amazon in the United States, Taobao in China, etc.) have recently launched a new form of online reviews called question-and-answer reviews (i.e., Q&A reviews). In Q&A reviews, potential consumers can ask customized questions on target products to those already-purchased consumers. Q&A reviews featured with social Q&A provide direct interactions (Lee and Brusilovsky, 2017), which in turn can enhance consumers’ trust to e-commerce platforms (Nadeem et al., 2020) and perceived usefulness (Guan et al., 2018).

However, although Q&A reviews have the mentioned merits, engagement in Q&A reviews is still not active enough. For example, the answering rate of iPhone 13 pro in JD.com (a popular e-commerce platform in China) was only around 50% by the time we finished this paper. Q&A reviews are totally voluntary and non-mandatory, no party can guarantee that every question will have a corresponding answer (Guan et al., 2018; Fang and Zhang, 2019). Consumers are generally social loafers rather than contributors, thus most of them are not likely to post reviews online (Yan et al., 2018). To increase consumers’ intention to Q&A reviews and boost consumer engagement, we must comprehend what motivates this intention. Understanding consumers’ motivations to share Q&A reviews, not only contributes to the researches of eWOM and consumer engagement, but also can help practitioners encourage consumer’s online knowledge sharing, which in turn can affect consumers’ loyalty or commitment to platforms (Dessart et al., 2019).

Q&A reviews not only involve the information of target products, but other extensive matters (e.g., comparisons of substitutes, etc.). Therefore, according to the contents and functions of Q&A reviews, we regard consumers’ intention to answering in Q&A reviews as consumer knowledge-sharing intention. But unfortunately, to our knowledge, there is no research examining online reviews from the perspectives of social Q&A or knowledge-sharing intentions. Generally, social Q&A is mostly discussed in the context of online communities (Neshati et al., 2017), whereas knowledge-sharing are discussed in corporate organizations (e.g., Matsuo and Aihara, 2022; Nguyen et al., 2022). Even studies on eWOM, little research have empirically investigated the motivations for consumers to write online reviews in e-commerce (Nam et al., 2020). Previous studies generally focus on relationships between specific variables and eWOM, such as satisfaction (Thakur, 2019; Singh and Söderlund, 2020), brand love (Bairrada et al., 2018), brand attitude (Izogo and Mpinganjira, 2021) and financial incentives (Qiao et al., 2020), or focus on the motivations to read eWOM (e.g., Teng et al., 2017; Ismagilova et al., 2020; Mainolfi et al., 2022). Q&A reviews and their motivations still lack empirical studies. Besides, although Q&A reviews involve two parties (i.e., questioners and answerers), we only discuss knowledge-sharing intention from the perspective of answerers, since questioners’ motivations are relatively obvious.

This study starts with the fundamentals of social Q&A and eWOM to specify the concepts of Q&A reviews and consumer knowledge-sharing intention. Then, based on self-determination theory, we explore the motivations to consumers’ knowledge-sharing intention. Moreover, this study also discusses the outcomes of consumer knowledge-sharing and the moderation role of feedback (i.e., comments and likes). In this study, we define feedback as a post-replying behavior, i.e., behavior of responding to the answers. Feedback is a key function and an important social interaction indicator in Q&A reviews, but currently few studies have examined this topic (Fang et al., 2018). Finally, according to the results of empirical tests, this study provides some suggestions for future research on knowledge-sharing and social Q&A and shed light on practices of e-commerce platforms.

## Literature Review

### Q&A Reviews and Consumer Knowledge-Sharing

Although Q&A review is a special type of online reviews, it more attributes to social Q&A when considering its design features. For example, potential consumers can ask questions related to target products in Q&A reviews. Then those already-purchased consumers will receive invitations, which randomly sent by e-commerce platform systems, and decide whether or not to answer the questions. Finally, other consumers can give feedback (e.g., likes or comments) to those answers. Therefore, in this study we use consumer knowledge-sharing intention to reflect consumers’ answering intention in Q&A reviews.

Q&A reviews generally have the following advantages. First, Q&A reviews can help consumers to improve efficiency (Shah and Kitzie, 2012). Information on the internet is always overloading and disordered. Traditionally, consumers have to spend extra time in reading numerous sources of information and finding answers on online reviews, but in Q&A reviews potential consumers can access this demanding through direct interaction. The cognitive pressure from information integration also can be reduced (Bleda et al., 2021). Second, Q&A reviews can help consumers to improve the perception of credibility and usefulness. Q&A reviews that provide accurate information reduce perceived uncertainty (Reimer and Benkenstein, 2016). For example, potential consumers can ask already-purchased consumers about specific and individual questions. Moreover, in traditional online reviews, any consumers can provide their evaluations. But as the rules of Q&A reviews, when questioners post their questions, e-commerce platforms will send random invitations to some of the consumers who have already purchased the products. Then those eligible consumers can give their answers voluntarily. In this sense, some unfairly negative reviews can be filtered, and certain perceived risks can be reduced. Third, Q&A reviews realize direct interaction between consumers and thereby satisfy their social needs (Jin et al., 2015). Not only can questioners receive responses from answerers, but answerers can get feedbacks (e.g., comments and likes) from other consumers.

### Motivations

#### Internal Motivations

The participation rate in Q&A reviews is relatively low. It is necessary to analyze consumers’ motivations to increase their participation intention (Hajli and Lin, 2016), since motivation is an important factor that drives individual behaviors. However, little research have investigated the factors motivating consumers to write eWOM (Nam et al., 2020). Due to the fact that Q&A reviews are featured with social Q&A and are different to traditional online reviews, this research examines the consumers’ motivations to answer questions in Q&A reviews not only from the perspective of eWOM, but also from social Q&A and online knowledge sharing.

Several studies on online knowledge sharing (e.g., Chen et al., 2019b; Kang, 2022) show that the theoretical framework of self-determination theory is useful in explaining motivations. In self-determination theory, motivations can be divided into internal motivations and external motivations. Internal motivations refer to the motivations in which behaviors are driven by individuals’ own interests or pleasures, without caring whether there are rewards or not (Deci and Ryan, 1990).

Altruistic motivation is one key internal motivation that drives individuals to share knowledge (Ismagilova et al., 2021). Altruistic behavior is voluntary and performed by the individuals with clear awareness and those behaviors must be beneficial to others, however, the conductors can only receive a feeling of internal self-reward (e.g., “a feeling of doing good”), without expecting any spiritual or material returns (Trivers, 1971; Bar-Tal, 1986). From human nature, people intrinsically have the altruistic motivation to help others without return (Wasko and Faraj, 2005). Especially after purchasing, consumers are always willing to share their experiences to help others to make decisions (Ismagilova et al., 2021). Previous literature has highlighted the important role of altruistic motivations in knowledge sharing. For example, in the context of social Q&A, Fang and Zhang (2019) found the answerers in a social Q&A platform had altruistic motivations to give their answers. Yuan and Liu (2017) found altruistic motivations have significant positive influence on persistent sharing willingness. Similar results were also found in eWOM (e.g., Hu and Kim, 2018; Liu et al., 2020; Chai et al., 2022).

Therefore, we predict that the altruistic motivation of helping others is one motivation to make consumers would like to share knowledge in Q&A reviews:

H1: *Consumers’ altruistic motivations positively influence consumers’ knowledge-sharing intention.*

Self-determination theory suggests three fundamental needs: autonomy, competence and relatedness—that are “the basis for self-motivation and personality integration, as well as for the conditions that foster those positive processes” (Ryan and Deci, 2000, p. 68). When satisfied with three fundamental needs, individuals can perceive enjoyment (Reinecke et al., 2014) and be driven to engage in knowledge sharing. The design features in Q&A reviews are closely related to these three needs. For example, as Q&A reviews allow consumers answer questions voluntarily, the need for autonomy would be enhanced. Furthermore, consumers may be challenged by questions or others’ feedback in Q&A reviews. However, when they solve those questions and get positive feedback, they may perceive the need for competence (Cui and Ji, 2019). Finally, Feedback is an important social interaction indicator (Fang et al., 2018). In Q&A reviews, the answers can get others’ feedback, such as comments or likes, may perceive the need for relatedness.

Therefore, we posit that consumers would be motivated by hedonism to share knowledge in Q&A reviews. Past studies (e.g., Jin et al., 2013; Zhao et al., 2016) have indicated that the reasons for consumers to produce word-of-mouth (WOM) are always sentimental (Kähr et al., 2016). Generally, consumers are willing to share their knowledge about brands, products or other consumption experiences to support their favorite brands. For example, through systematic review, Rosario et al. (2020) identified hedonic motivations are important for consumers to create eWOM. Empirical evidence from Nam et al. (2020) indicated that hedonic motivations significantly predicted in both positive and negative eWOM. Similar results are also provided by research on social Q&A and knowledge sharing. For example, both Zhao and Detlor (2021) and Wang et al. (2022) found users were intrinsically motivated by hedonism to share knowledge in online communities. In social Q&A, Zhao et al. (2016) also conducted a study to prove that the enjoyment in helping others is the main reason for users to share their knowledge. To sum up, consumers can feel enjoyment when they share their knowledge (Yun et al., 2007):

H2: *Consumers’ hedonic motivation positively influence consumers’ knowledge-sharing intention.*

On the contrary, people are driven by external motivations for separable outcomes, such as gaining some external rewards, avoiding certain punishments or reducing certain pressures (Ryan and Deci, 2000). When motivated externally, individuals tend to obtain instrumental value from participating, rather than enjoying the activity itself. However, due to some reasons of self-regulations, external motivations can be internalized into internal motivations (Mitchell et al., 2020).

Since online communities vary in platform characteristics and affordances, there is a need to explore different factors in affecting eWOM creation (Rosario et al., 2020). In Q&A reviews, after someone ask a question, the e-commerce platform system will randomly send invitations to consumers who have already purchased the products. Besides, if those invited consumers choose to answer, they will gain certain point rewards. Therefore, according to self-determination theory and the design features and rules of Q&A reviews, we predicted that the consumers’ external motivations for knowledge-sharing are perceived pressure and external rewards.

#### Perceived Pressure

Individuals generally perceive pressure from social subjective norms, which refers to that when people find someone are important to them, they may thus be influenced to perform certain behaviors (Choi et al., 2020) or motivated to comply others’ opinions (Ham et al., 2015). Invitations are core operation mechanism of Q&A reviews. E-commerce platform systems randomly send invitations to already-purchased consumers. Consumers may find other potential consumers are in need of help, and thus perceive pressure to share their knowledge on products. Moreover, Guan et al. (2018) indicated that in social Q&A, when individuals turn to others for help or get help from others, they are constrained by norms and perceive pressure from reciprocity to share knowledge. Therefore, it is necessary to investigate the role of perceived pressure in motivating consumers to answer questions.

Although little research has studied the influence of perceived pressure on knowledge sharing intentions in both eWOM and social Q&A, research in online knowledge sharing has provided some evidence. For example, Zhou (2021) found users in online health communities are motivated by social subjective norms to share knowledge. Choi et al. (2020) compared two different online mobile platforms and found subjective norms significantly influenced users’ intention to share knowledge in both platforms. Therefore, in this study we hypothesized:

H3: *Consumers’ perceived pressure positively influence consumers’ knowledge-sharing intention.*

#### External Rewards

Generally, in the context of eWOM, external rewards are generally related to economic incentives, such as bonus gifts or airline miles for upgrades, which can motivate consumers to generate eWOM (Yoo et al., 2013). However, in online communities, there is no economic incentives systems, but platforms often use community points/badges/rankings instead to motivate users’ knowledge sharing intentions (Zhang et al., 2017).

Some research suggests the positive relationship between virtual rewards and knowledge sharing. For instance, Zhao et al. (2016) found virtual rewards can incentive inactive users to share knowledge in social Q&A sites. Wang et al. (2022) further indicate virtual rewards have strong positive influence on explicit knowledge sharing. In terms of Q&A reviews, people can get points through answering questions. The points not only reflect an individual’s social status or reputation but can help people to obtain exclusive discounts when their points reach a certain level. Therefore, in this study we hypothesized:

H4: *External rewards positively influence consumers’ knowledge-sharing intention.*

### Loyalty to Platform

In this article, we regard loyalty to platform as a consumer’s intention to repeat visiting an e-commerce platform (Cyr et al., 2005). Loyalty to platform reflect the level of consumer preference for a certain e-commerce platform (Jones and Taylor, 2012). Yao et al. (2015) conducted a field survey of 222 online community members and found knowledge-sharing was positively related to members loyalty. Similar conclusion was presented by Swanson et al. (2020), although they discussed the topic within organizations.

In this study, we predicted that consumers’ knowledge-sharing intention will positively affect consumers’ loyalty to platform based on the following reasons: First, the process of Q&A reviews contains social capital, and social capital can affect consumer loyalty (Jones and Taylor, 2012). The knowledge shared by consumers can be regarded as representing the utilitarian values (i.e., social capital) of e-commerce platforms and thus lead to higher loyalty to the platforms (Wang and Fesenmaier, 2004a). Second, social interaction will directly or indirectly affect consumer loyalty to platforms (Koh and Kim, 2004). Consumers’ knowledge-sharing is a kind of direct social interaction between consumers. These interactions can induce consumers’ social sentiments and make them feel as they are talking to real people in person. Moreover, consumers also tend to deem the platform as an enthusiastic, generous and social place, and are more willing to maintain the relationship with the platform (Nadeem et al., 2020). Finally, after sharing knowledge, consumers will have a sense of responsibility and ownership toward the platform, and in turn induce a higher level of loyalty (Yao et al., 2015). Therefore, in this study we hypothesized:

H5: *Consumers’ knowledge-sharing intention positively influence consumers’ loyalty to platform.*

In addition, we also take an initial step on assessing the moderator role of feedback between variables. In this study, we define feedback as a post-replying behavior, i.e., behavior of responding to the answers. Feedback is a key function and an important social interaction indicator in Q&A reviews, but currently few studies have examined this topic (Fang et al., 2018). Traditionally, consumers who write online reviews cannot obtain feedback from others, but in Q&A reviews, other consumers or sellers can express their opinions to answerers using the “like” or “reply” functions. This feedback mechanism can further deepen the degree of social interaction on e-commerce platforms and increase consumer engagement and loyalty (Jin et al., 2015; Guan et al., 2018).

In this study we predicted that feedback (i.e., likes and comments) can moderate the relationship between consumers’ motivations and knowledge-sharing intention. Firstly, Q&A reviews are actually a form of social interaction. If consumers are motivated to share knowledge, it means that they have a need for social interaction. Feedback is a key indicator of social dynamics (Bateman et al., 2011) and can be regarded as a dynamic communication process between consumers. Therefore, we believe that feedback can satisfy consumers’ social interaction needs and thus promote higher levels of participation (Stavrositu and Sundar, 2012). Secondly, feedback not only can enhance the willingness of people to share knowledge (e.g., Brzozowski et al., 2009), but also is a form of motivational affordance, which may affect consumers’ intrinsic motivations (e.g., Fong et al., 2019). Even more, Chen et al. (2019a) explored the interaction effect of two motivational affordances (i.e., votes and comments) and found the number of comments (which can be seen as feedback) moderated the relationship between usefulness voting and knowledge contribution.

In Q&A reviews, feedback usually takes the form of a “like” or a “comment.” As a positive form of feedback, likes can satisfy consumers’ self-competence needs and improve their level of intrinsic motivations (Fong et al., 2019), whereas comments are a direct means of social communication that can increase perceived involvement and perceived social presence, and further lead to a higher level of affective experience (Fortin and Dholakia, 2005), which in turn can influence consumers’ internal motivations and promote them to induce continuous intentions on knowledge-sharing. However, in terms of the external motivations in Q&A reviews, they mainly come from the pressure of potential consumers (or sellers), or from the rewards provided by the platform. External elements will not change along with the number of feedback responses. Therefore, we hypothesized that:

H6a: *Feedback moderate the relationship between an altruistic motivation and knowledge-sharing intention. The influence of an altruistic motivation on knowledge-sharing intention is stronger under conditions in which feedback is received (vs. non-feedback conditions).*

H6b: *Feedback moderate the relationship between a hedonic motivation and knowledge-sharing intention. The influence of a hedonic motivation on knowledge-sharing intention is stronger under the conditions in which feedback is received (vs. non-feedback conditions).*

Moreover, in this study we propose that feedback can also moderate the relationship between consumers’ knowledge-sharing intention and loyalty to platform. Consumers who participate in knowledge-sharing can perceive utilitarian value, hedonic value, and social value through receiving feedback (Wang and Fesenmaier, 2004b). Those values may result in the sense of mutual benefit and consumers are thus more willing to use the e-commerce platform. For example, as a positive feedback, a “like” can enable answerers to confirm their values and make them feel social support and a sense of belonging (Chiu et al., 2006). Consumers may be more willing to participate in platform activities (Guan et al., 2018). On the other hand, the social interaction involved in feedback can increase consumers’ perceived intimacy toward the platform (Hassanein and Head, 2007), and additionally, consumers may also perceive social capital in the feedback they receive (Son et al., 2016). The content of the comments (i.e., feedback) generally supplements the incomplete information provided by original answerers or provides further discussion of the question. Comments can expand the level of knowledge and make the answerers perceive utilitarian values.

Therefore, these values may make consumers who participate in knowledge-sharing more likely to have positive attitudes toward the platform (Chung and Buhalis, 2008) and increase loyalty to platform. Thus, we hypothesized:

H6c: *Feedback may moderate the relationship between knowledge-sharing intention and loyalty to platform. The influence of knowledge-sharing intention on loyalty to platform is stronger under conditions in which feedback is received (vs. non-feedback conditions).*

**FIGURE 1:**
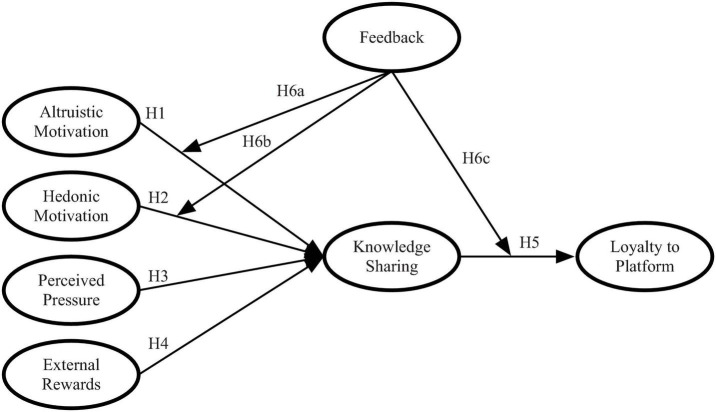
Hypothesized model.

The hypothesized model is illustrated in Figure 1.

## Methodology

To test our hypotheses, we recruited participants on a research data platform^1^ to collect data in exchange for monetary compensation. The questionnaires were randomly distributed to consumer panel by the platform system.

Taobao is one of the biggest e-commerce platforms with a considerable number of consumers (or users) in China. It was among the first e-commerce platforms to launch Q&A reviews to help consumers solve their pre-purchase decision-making problems through direct interaction in the year 2015. The mechanism of the Q&A reviews in Taobao has developed over time, attaining a relatively more mature status in recent years. Therefore, we asked participants if they ever shared knowledge in Q&A reviews in Taobao and excluded the data who did not have the knowledge-sharing experiences.

In order to avoid common method biases, this study involved prior controls in the process of the questionnaire setting, according to Podsakoff et al. (2003). First of all, the scales in this research were adapted from the extant scales and were rated on 7-point Likert scale (1 = strongly disagree, 7 = strongly agree). For example, the altruistic motivation scale was adapted from Hennig-Thurau et al. (2004); Yoo et al. (2013) and Munar and Jacobsen (2014); the hedonic motivation scale was adapted from Childers et al. (2001), containing two reverse-coded items; the perceived stress scale was adapted from Picazo-Vela et al. (2010); the external rewards scale was adapted from Hennig-Thurau et al. (2004); Yoo et al. (2013); the analysis of consumers’ online knowledge-sharing intention was adapted from Lai and Chen (2014); and the loyalty to platform scale was adapted from Kim and Ahn (2017). The specific items are provided in the appendix. Feedback, as a moderating variable, was measured as a binary variable (1 = feedback-received; 0 = non-feedback). Specifically, participants were asked whether they had received replies or likes after answering the questions. Secondly, all the arranged items and constructs were presented in a random order. Finally, we instructed the participants that the survey was anonymous and private, and all the answers were correct in order to diminish some considerations.

As a result, we collected a sample of 255 data. A total of 112 were males and 143 were females. The majority of the sample (118 persons, 46.27%) were aged between 26 and 30, 56 (21.96%) participants were younger than 25, 72 (28.24%) were aged between 31 and 40, and 9 (3.53%) were older than 41 years.

## Results

Following the two-steps approach recommended by Anderson and Gerbing (1988), we analyzed the data using R SEM package lavaan (Rosseel, 2012). Though the data were not multivariate normal distributed, we still used maximum likelihood estimation because previous research suggests this method is robust even when the multivariate normality assumption is violated (Bollen, 1989; Diamantopoulos et al., 2000). We first report the measurement model results, and then we report the structural model results. In order to test the moderating role of feedback, we did an additional multigroup analysis.

### Measurement Model Evaluation

Although the measurement model yielded a significant chi-square (χ^2^ = 536.95, *df* = 260, *p* < 0.05), the ratio of chi-square to degree of freedom (χ^2^/*df* = 2.07) was with the acceptable range of 2–5 (Marsh and Hocevar, 1985). Other fit statistics were also acceptable (GFI = 0.90, CFI = 0.92, AGFI = 0.90, TLI = 0.91, NNFI = 0.91, RMSEA = 0.06, SRMR = 0.05). This evidence set the foundation for the evaluations of the psychometric properties of the measurements.

In Table 1, we report the psychometric properties of the measurement model. We used several criteria to assess reliability and convergent validity. First, all Cronbach’s alphas of the measures for the latent constructs were larger than 0.70, indicating sound internal consistency reliability. Second, we calculated composite reliability using formula suggested by Fornell and Larcker (1981). The values of composite reliability ranged from 0.76 to 0.91, all were larger than 0.60, provided additional evidence for reliability (Bagozzi and Yi, 1988). Then, we calculated average variance extracted, the value ranged from 0.51 to 0.68, all were larger than 0.50, representing stricter evidence for convergence and reliability (Fornell and Larcker, 1981; Bagozzi and Yi, 1988). Finally, the factor loadings were assessed. All factor loadings were significant, and nearly all R^2^ were larger than 0.50, representing an indication for convergent validity (Bagozzi et al., 1991).

**TABLE 1:** Items, factors loadings, and reliability estimates.

Latent variables	Items	St. Factor Loadings	Cronbach’s α	AVE	CR
Altruistic Motivation	I want to help others find a great product through Q&A reviews.	0.73	0.86	0.51	0.86
	I want to point out a good offer to others through Q&A reviews.	0.72			
	I like to help others with advice through Q&A reviews.	0.73			
	I want to support an online shop or brand that I like through Q&A reviews.	0.69			
	I really like the product or shop and want to contribute to its success through Q&A reviews.	0.76			
	I look forward to the product and want to reward the online shop or the brand through Q&A reviews.	0.68			
Hedonic Motivation	Sharing knowledge on Q&A reviews would make me feel good.	0.79	0.91	0.61	0.91
	Sharing knowledge on Q&A reviews would be boring.	0.74			
	Sharing knowledge on Q&A reviews would involve me in the shopping process.	0.80			
	Sharing knowledge on Q&A reviews would be exciting.	0.78			
	Sharing knowledge on Q&A reviews would be enjoyable.	0.86			
	Sharing knowledge on Q&A reviews would be uncomfortable.	0.73			
	Sharing knowledge on Q&A reviews would be interesting.	0.72			
Perceived Pressure	I feel pressure from intermediary (e.g., Taobao) to share knowledge on Q&A reviews	0.65	0.76	0.52	0.76
	Sellers expect that I share knowledge on Q&A reviews	0.76			
	Future potential customers anticipate that I share knowledge on Q&A reviews	0.75			
External Rewards	I want to get points through Q&A reviews.	0.77	0.83	0.62	0.83
	I want to get a discount through Q&A reviews.	0.81			
	I can save money on my next purchase through Q&A reviews.	0.79			
Knowledge-sharing Intention	If I had some knowledge about a topic, I would consider posting it on Q&A reviews	0.85	0.80	0.68	0.80
	If I had some knowledge regarding a question someone asked, I would share this knowledge with others	0.80			
Loyalty to platform	The intermediary (e.g., Taobao) where I provide Q&A reviews is always my first choice to shop.	0.77	0.87	0.68	0.87
	I always visit the intermediary (e.g., Taobao) where I provide Q&A reviews.	0.83			
	I usually consume on the intermediary (e.g., Taobao) where I provide Q&A reviews.	0.77			
	Overall, I am loyal to the intermediary (e.g., Taobao) where I provide Q&A reviews.	0.81			

We tested discriminant validity using method suggested by Fornell and Larcker (1981) that a scale possesses discriminant validity if the square root of average variance extracted by the underlying construct is larger than the correlations with other latent constructs. As shown in Table 2, each square roots of average variance extracted of latent constructs were larger than the correlations between possible pairs of latent constructs, thus provided supports for discriminant validity between the measures of the constructs.

**TABLE 2:** Descriptive statistics, correlations and discriminant validity statistics.

	Altruistic motivation	Hedonic motivation	External reward	Perceived pressure	Knowledge sharing intention	Loyalty to platform
Altruistic Motivation	**0.71**					
Hedonic Motivation	0.58	**0.78**				
External Reward	0.36	0.30	**0.79**			
Perceived Pressure	0.57	0.49	0.45	**0.72**		
Knowledge Sharing Intention	0.57	0.55	0.41	0.53	**0.82**	
Loyalty to Platform	0.64	0.41	0.30	0.60	0.44	**0.82**
Mean	5.67	5.26	5.30	5.0	5.85	5.55
SD	1.06	1.24	1.33	1.42	1.07	1.21

*The diagonal line is the square root of AVE of each construct; the lower triangle is the correlation coefficients between constructs; p < 0.01.*

In addition, we used Harman’s single-factor test to check for potential common method biases. All the indicators of the constructs were incorporated in the unrotated exploratory factor analysis. The result revealed that a single-factor only explained 34% of the variance, indicating no “general” factor in the data thus common method biases was not a big concern (Podsakoff and Organ, 1986).

In summary, the measurement model results demonstrated satisfactory psychometric properties required to proceed to the structural model evaluation and hypotheses testing.

### Hypotheses Testing

We first tested our hypothesized conceptual model without incorporating the moderating variable. The path model revealed an acceptable fit despite of a significant chi-square (χ^2^ = 602.22, *df* = 264, *p* < 0.05; χ^2^/*df* = 2.28; GFI = 0.90, CFI = 0.90, AGFI = 0.90, TLI = 0.90, NNFI = 0.90, RMSEA = 0.06, SRMR = 0.05).

**FIGURE 2:**
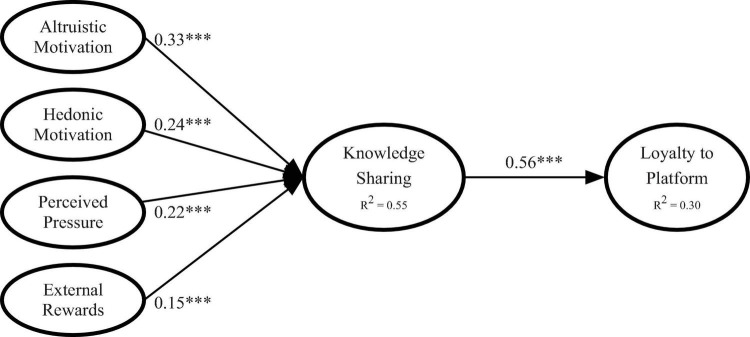
Path coefficients of the structural model. ^***^Denotes *p* < 0.01.

Figure 2 illustrates the standardized path coefficients of our proposed relationships. The results revealed that altruistic motivation (β = 0.33, *p* < 0.01), hedonic motivation (β = 0.24, *p* < 0.01), perceived stress (β = 0.22, *p* < 0.01), and external rewards (β = 0.15, *p* < 0.01) had significant positive effects on consumers’ knowledge-sharing intention. H1–H4 were all supported. H5, postulating a positive relationship between knowledge-sharing intention and loyalty to platform, was also supported (β = 0.56, *p* < 0.01).

To test the hypotheses of the moderating role of feedback, we split the sample to two groups according whether the respondents reported they got feedback or not after they provided answers to user generated questions (N_feedback_ = 213; N_no–feedback_ = 42). We then compared three multigroup confirmative factor models to ensure the multigroup measurement invariance. For the first model, the same factor structure was imposed on all groups without constraining the factor loadings and intercepts to be equal across groups (χ^2^ = 1,028.57, *df* = 520); for the second model, not only the factor structure, but also the factor loadings were constrained to be equal across (χ^2^ = 1,052.99, *df* = 539); for the third model, the factor structure, the factor loadings, and the intercepts were all constrained to be equal across groups (χ^2^ = 1,081.09, *df* = 558). Chi-square difference test indicated that there was no significant difference between the first model and the second model (Δχ^2^ = 24.42, Δ*df* = 19, *p* = 0.18), and there was no significant difference between the second model and the third model (Δχ^2^ = 28.10, Δ*df* = 19, *p* = 0.08), indicating strong evidence for measurement invariance across groups in terms of both metric invariance and scalar invariance (Putnick and Bornstein, 2016). Thus, measurement invariance across groups enables us to test the moderating role of feedback using multigroup path analysis.

Following procedures used in Homburg et al. (2005), we tested each of the moderated paths one by one by comparing two models, one model constrained all paths to be equal across groups (no-moderation model), and the other constrained all other paths to be equal except for the one hypothesized moderated path. We relied on chi-square difference test to examine whether the latter model was significantly better than the former model. If the chi-square improvement for the latter model is significant, that is evidence for the moderating hypothesis. As Table 3 shows, H6a and H6c were supported, H6b was not supported.

**TABLE 3:** Results of multigroup analysis.

Hypothesized moderated paths	Feedback		Δχ^2^ (Δ*df* = 1)
	Yes	No	
H6a: Altruistic Motivation - > Knowledge Sharing Intention	0.54*	−0.07^n.s.^	9.87*
H6b: Hedonic Motivation - > Knowledge Sharing Intention	0.40*	0.19*	1.42^n.s.^
H6c: Knowledge Sharing Intention - > Loyalty to Platform	0.71*	−0.10^n.s.^	10.39*
No-moderation model: χ^2^ = 1134.71, *df* = 533			

*Denotes p < 0.01; n.s. denotes non-significance.

## General Discussion

Although eWOM and traditional online reviews are important for consumer decisions, they may also have some shortcomings (e.g., perceived problems with their trustworthiness or usefulness). On the contrary, with direct social interactions, Q&A reviews are probably capable of conquering those regrets and benefiting consumer loyalty. However, little research empirically investigates the motivations to provide eWOM (Nam et al., 2020). Especially, there is no research examining online reviews from the perspectives of social Q&A or knowledge-sharing intentions. We are the very first research to explore Q&A reviews, and related antecedences and consequences. Understanding consumers’ knowledge-sharing intentions in Q&A reviews has important theoretical and practical implications.

### Theoretical Implications

Q&A reviews are different to traditional online reviews. They are featured with social Q&A. Therefore, we examined Q&A reviews not from the perspective of eWOM, but also from social Q&A and knowledge sharing. Prior research on social Q&A are mostly in the context of online communities (Neshati et al., 2017), whereas knowledge-sharing are discussed in corporate organizations (e.g., Matsuo and Aihara, 2022; Nguyen et al., 2022). Our results extend the research perspectives of eWOM and contribute multidisciplinary to both eWOM and social Q&A.

Firstly, based on self-determined theory and the results of our empirical research, we found that consumers’ motivations for knowledge-sharing intention in Q&A reviews consist of altruistic motivations, hedonic motivations, perceived pressure, and external rewards. Among these, the former two motivations are internal, and the latter two are external. Previous studies seldom examine consumers’ motivations to write eWOM, but generally focus on relationships between specific variables and eWOM, such as satisfaction (Thakur, 2019; Singh and Söderlund, 2020), brand love (Bairrada et al., 2018), brand attitude (Izogo and Mpinganjira, 2021) and financial incentives (Qiao et al., 2020), or merely focus on the motivations to read eWOM (e.g., Teng et al., 2017; Ismagilova et al., 2020; Mainolfi et al., 2022).

Secondly, previous studies discussed the relationship between knowledge-sharing intention and loyalty either in the context of online communities (e.g., Yao et al., 2015) or organizations (e.g., Swanson et al., 2020). However, our results firstly shed light on the influence of consumers’ knowledge-sharing intention on consumers’ loyalty to platform in the context of e-commerce. Since Q&A reviews attribute to social Q&A, consumers may perceive a greater social presence, social interactions, and higher level of ownership from platforms, and thus perform higher level of loyalty.

Thirdly, we also highlight the moderation role of feedback. We define feedback as a post-replying behavior, i.e., behavior of responding to the answers. Feedback is a key mechanism of Q&A reviews. Our empirical results show that when feedback is received, consumers’ altruistic motivations have a stronger influence on knowledge-sharing intention and their knowledge-sharing intentions have a stronger influence on loyalty to platform. Although feedback did not significantly moderate the relationship between hedonic motivations and knowledge sharing intention, the evidence presented in this research still provides a new perspective of feedback, since seldom studies have examined it (Fang et al., 2018).

### Managerial Implications

According to the results of our research, we found altruistic motivations, hedonic motivations, perceived pressure, and external rewards significantly influence consumers’ knowledge sharing intentions. Due to the fact that altruistic motivation is more related to personality characteristics, we suggest managers to stimulate other three motivations. For instance, practitioners can stimulate consumers’ hedonic motivations through gamify Q&A reviews. Hamari and Koivisto (2015) has pointed out that the hedonic value is the key value that consumers can perceive from gamified products or services. Gamification is more often used in a marketing context. Therefore, adding gamification elements may improve consumers’ hedonic motivations to participate in knowledge-sharing.

Besides, as perceived pressure derived from social norms, managers need to present consumers’ values and others’ active participation intentions in knowledge sharing (Zhou, 2021). Moreover, past studies suggested that external motivations can be internalized *via* gamification (Bittner and Schipper, 2014; Kuo and Chuang, 2016). Managers may thus gamify external rewards to realize internalization and increase the impact of extant motivations. For example, Kim and Ahn (2017) found that internal motivation has a strong positive relationship with consumer engagement and external motivation can be internalized by applying game elements to increase consumer engagement. Therefore, after consumers share their knowledge, practitioners may present some games (e.g., lottery, competitions, etc.) not only to make them gain some rewards, but also to satisfy their hedonic needs.

On the other hand, we found that feedback has a significant moderating effect. When receiving feedback, consumers’ altruistic motivations may have stronger influences on knowledge-sharing and in turn has a stronger influence on their loyalty to platform. Managers may set notification function of feedback, or use virtual external rewards (e.g., points, badges) to encourage consumers to provide more feedback and thus enhance social dynamism of e-commerce platforms.

### Limitations and Future Research Directions

Due to the limitation of time, we only collected data of 42 respondents who did not get feedback. The sample size problem might be the reason why some of the paths were not significant. Future studies may collect more data of people who did not get feedback and test the moderation effect of the relationship between internal motivation and knowledge sharing intentions.

Moreover, Future studies can further explore other motivations behind consumers’ knowledge-sharing intention. For example, social recognition is another possible motivation. Research in organizations has shown that the purpose of employees in contributing knowledge is to gain recognition from other colleagues (Constant et al., 1996); furthermore, in the context of social Q&A, research (e.g., Rui and Whinston, 2012) indicates that users expect to gain the attention of others by sharing knowledge, and they may even write comments that cater to other users’ interests to gain more social recognition (Fang et al., 2018). Therefore, in the context of e-commerce, consumers may also have expectations of winning social recognition from others, and this can be regarded as one motivation.

Finally, future research may place more of a focus on feedback. In this study, feedback referred to the behavior of providing comments or likes. Although responding behavior is an important social interaction indicator and feedback is also an important mechanism in Q&A reviews, there is still a lack of studies discussing this topic (Fang et al., 2018). In this research, we highlight the moderation role of feedback. However, in Q&A reviews, feedback from consumers is also spontaneous and voluntary. Hence, to exploit the critical impacts of feedback, future studies may further discuss its antecedents. Finally, in terms of outcome variables, as loyalty to platform reflects consumers’ willingness to repurchase on e-commerce platforms, second-hand data can be used in future studies to further discuss the relationship between consumer knowledge-sharing intention and repurchase frequency or other related variables.

## Data Availability Statement

The raw data supporting the conclusions of this article will be made available by the authors, without undue reservation.

## Author Contributions

All authors contributed to literature review, methodology, survey, data analysis, writing, and have read and agreed to the published version of the manuscript.

## Conflict of Interest

The authors declare that the research was conducted in the absence of any commercial or financial relationships that could be construed as a potential conflict of interest.

## Publisher’s Note

All claims expressed in this article are solely those of the authors and do not necessarily represent those of their affiliated organizations, or those of the publisher, the editors and the reviewers. Any product that may be evaluated in this article, or claim that may be made by its manufacturer, is not guaranteed or endorsed by the publisher.

## Footnotes

1
http://credamo.com
